# Virus-like particle (VLP)-based indirect ELISA (iELISA) for the detection of beak and feather disease virus (BFDV) antibodies

**DOI:** 10.1007/s00253-025-13683-z

**Published:** 2026-01-23

**Authors:** Pangkaj K. Dhar, Tridip Das, Babu K. Nath, Prabal Chowdhury, Andrew Peters, Jade K. Forwood, Shane R. Raidal, Shubhagata Das

**Affiliations:** 1 School of Agricultural, Environmental and Veterinary Sciences, Charles Sturt University, Wagga Wagga, NSW-2678 Australia; 2 Training Hub Promoting Regional Industry and Innovation in Virology and Epidemiology, Gulbali Institute, Charles Sturt University, Wagga Wagga, NSW-2678 Australia; 3 Biosecurity Research Program and Training Centre, Gulbali Institute, Charles Sturt University, Wagga Wagga, NSW-2678 Australia; 4 Asia Pacific Centre for Animal Health, Melbourne Veterinary School, Faculty of Science, The University of Melbourne, Parkville, VIC-3010 Australia; 5 Melbourne Veterinary School, Faculty of Science, The University of Melbourne, Werribee, VIC-3030 Australia

**Keywords:** Indirect ELISA, BFDV, Serodiagnosis, Psittacine, TG-ROC

## Abstract

**Abstract:**

Beak and feather disease virus (BFDV) poses a significant threat to avian biodiversity and global aviculture. Reliable serodiagnostic tools are critical for assessing host immune status and guiding disease management. The haemagglutination inhibition (HI) assay, although historically regarded as the gold standard, is limited by technical complexity and its reliance on seropositive Galah erythrocytes, restricting broader application. This study describes the development and validation of a recombinant BFDV capsid protein-based indirect enzyme-linked immunosorbent assay (iELISA) for detecting anti-BFDV antibodies using dried blood spots from multiple psittacine species. The assay demonstrated high sensitivity and strong analytical performance, employing virus-like particles (VLPs) recombinantly expressed in *Escherichia coli.* Optimisation of the diagnostic cut-off by two-graph receiver operating characteristic (TG-ROC) analysis established an OD threshold of 1.73, achieving 96.5% sensitivity with a Youden’s index of 0.74. Discriminative capacity was further supported by receiver operating characteristic (ROC) analysis, yielding an area under the curve (AUC) of 0.896. Agreement with the HI assay was very strong (Gwet’s Agreement Coefficient 1= 0.843). This iELISA represents a scalable and universal serodiagnostic tool, supporting clinical diagnosis, enabling large-scale epidemiological investigations, and advancing conservation-focused BFDV surveillance.

**Key points:**

- *Efficient detection of anti-BFDV antibodies in psittacines using recombinant capsid protein as coating antigen.*
- *Use of TG-ROC curve to determine cut-off value supported by Gwet’s Agreement Coefficient 1.*
- *Easily adaptable method with very high sensitivity in detecting anti-BFDV antibodies.*

**Graphical Abstract:**

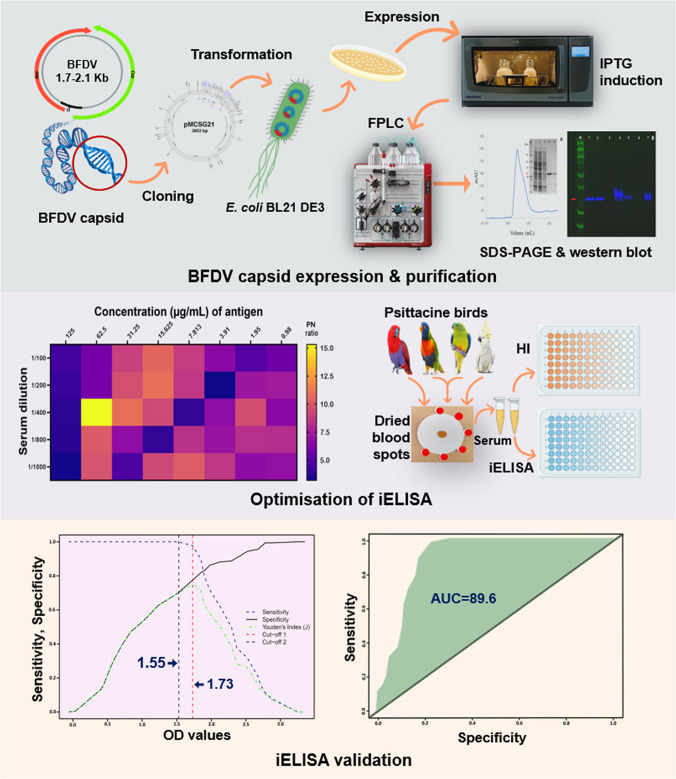

**Supplementary Information:**

The online version contains supplementary material available at 10.1007/s00253-025-13683-z.

## Introduction

Psittacine beak and feather disease (PBFD) is a widespread viral infection afflicting psittacine birds globally, caused by the beak and feather disease virus (BFDV), a member of the *Circoviridae* family (Pass and Perry 1984; Raidal and Cross 1995; Ritchie et al. 1989). The virus possesses a circular, single-stranded DNA (ssDNA) genome of 1.9–2.0 kb in length, encoding two major proteins: a capsid (Cap) protein, which plays a role in viral replication, attachment, and cellular entry and can self-assemble into icosahedral virus-like particles (VLPs) of 60 capsid protein molecules, and a replicase initiator (Rep) protein, which helps with viral replication (Bassami et al. 2001; Patterson et al. 2012; Ritchie et al. 1990; Sarker et al. 2016). BFDV disease exhibits a variety of clinical manifestations, ranging from acute infections in juveniles to chronic feather dystrophy in adult birds (Doneley 2003; Schoemaker et al. 2000).

BFDV Cap protomer’s agility to assemble as stable macromolecular structures when recombinantly expressed exalted it to form VLPs, which are remarkably compatible with serological diagnostic assays, including haemagglutination inhibition (HI). Despite lacking any established cell culture system, these recombinantly produced VLPs provide essential synthetic antigens in high concentration and purity for further downstream applications, including immunodiagnostics (Wang et al. 2020).

Existing diagnostic modalities for BFDV predominantly rely on molecular techniques such as polymerase chain reaction (PCR) and quantitative real-time PCR (qPCR), (Katoh et al. 2008; Shearer et al. 2009b; Ypelaar et al. 1999). Other molecular methods, like nested-PCR (Kiatipattanasakul-Banlunara et al. 2002), duplex shuttle PCR (Ogawa et al. 2005), and rolling circle amplification (Varsani et al. 2011), can be used in both diagnostic and research laboratories, offering valuable insights into viral load, strain variation, and infection dynamics. While these methods are sensitive for detecting active viremia, they cannot depict insight into the host’s immunological status or previous exposure.

Haemagglutination inhibition (HI) test, on the contrary, has emerged as the only reliable serological tool to assess antibody responses to BFDV in wild and captive birds, which is limited to certain laboratories (Raidal and Cross 1994; Raidal et al. 1993). HI antibody titers have been considered a strong negative predictor of PBFD in cockatoos (Khalesi et al. 2005; Ritchie et al. 1991), hence an active or persistent BFDV infection in other host species may exhibit low and fluctuating anti-BFDV HI titers. This may be the result of bursal or thymus damage and/or an effect of persistent infection in macrophages (Latimer et al. 1990). Moreover, HI assays may lack standardisation when native antigen is used, and therefore are impractical for high-throughput screening. Despite being considered the current gold standard, variability in virus preparation from infected birds, challenges associated with producing synthetic VLPs as an antigen, along with the availability of suitable erythrocytes, variable erythrocyte performance from individual birds, and variable sensitivities have hindered the adoption of HI testing from being an easily accessible, universal diagnostic tool (Sanada and Sanada 2000; Shearer et al. 2009a).

These identified factors underscore the need for a standardised, sensitive, and reproducible diagnostic alternative. Enzyme-linked immunosorbent test (ELISA) based on virus-like particles (VLPs) have become a strong candidate in veterinary diagnostics and surveillance of many infectious diseases and are also used to assess immunological responses to vaccination (Binns et al. 2007; Lequin 2005; Tang et al. 2013). Indirect ELISA (iELISA) offers a scalable, reproducible, and less labour-intensive alternative with the potential to detect past exposure and assess population-level immunity. This technique has been recommended as a standard tool for population-based serological studies (Wright et al. 1993). Use of dried blood spots (DBS) for serological analysis is well-established in serological diagnostics (Holroyd et al. 2022; Samsonova et al. 2022). DBS requires smaller blood volumes, simplifies storage and transportation without special treatment, and reduces biohazard risks (Malsagova et al. 2020; Sharma et al. 2014). This technique has been successfully applied in various fields, including pharmacokinetics, therapeutic drug monitoring, and disease diagnosis (Sharma et al. 2014). The existing pioneer in serodiagnosis of BFDV, HI test, also relies on DBS and produces excellent interpretation (Raidal et al. 1993). Moreover, BFDV from all psittacine bird species behave antigenically similarly, at least in haemagglutination and haemagglutination inhibition assays (Raidal et al. 1993). All these channelled the hypothesis of using DBS as a source of primary antibody in developing an indirect ELISA.

The present study was aimed at developing a robust, broadly applicable indirect ELISA (iELISA) using recombinantly expressed BFDV capsid VLPs, while prioritising validation of its diagnostic performance against the existing HI test. The assay demonstrated high sensitivity, specificity, and efficiency, thus presented as a reliable tool for clinical diagnosis, as well as for routine monitoring and surveillance.

## Materials and methods

### Blood samples

The study comprised 248 samples from four groups of psittacine birds: eclectus parrots, lorikeets, cockatoos, and orange-bellied parrots. All the samples had been tested as duplicates, and the mean OD value was counted for further analysis. Archived dried blood spots were retrieved randomly from the Veterinary Diagnostic Laboratory (VDL), Charles Sturt University, spanning 2023 and 2024. All of them were sent to VDL, aimed at tests other than iELISA. One punch of blood spot (6 mm) was chopped aseptically, rinsed in 400 µL PBS, vortexed, and incubated overnight at 4° C. The next day, serum was extracted by centrifuging at 10,000 RPM for 10 min. The resulting supernatant was carefully collected and stored at −20 °C until further analysis. A known anti-BFDV chicken serum sample was used as a positive control (Raidal et al. 1993), and a known chicken serum without any BFDV exposures was used as a negative control.

### Expression and purification of recombinant BFDV Cap protein

BFDV capsid gene was cloned into the *Ssp*l cloning site of the pMCSG21 vector, followed by transformation into *Escherichia coli* BL21 (DE3) Rosetta 2 cells (Novagen, Darmstadt, Germany) for recombinant expression (Sarker et al. 2015b). Starter culture was grown using these transformed colonies, and expression media was grown at 37 °C until an optimum OD_600_ of 0.4–0.6 was gained, then the temperature was shifted to 25 °C, and protein synthesis was induced by the addition of 0.5 mM isopropyl-β-D-thiogalactoside (IPTG) for up to 24 h of growth in total. The cells were then harvested through centrifugation in an Avanti JXN-26 (Beckman Coulter, Mt Waverley, VIC, Australia) at 6000 RPM for 30 min, and the cell pellets were resuspended in CAPS buffer A (20 mM *N*-cyclohexyl‐3-aminopropanesulfonic acid) (pH 10.5) and stored at ‐20°C.

By using FastBreak™ Cell Lysis Buffer (Promega, Alexandria, NSW, Australia) along with 20 mg lysozyme (Sigma‐Aldrich, St. Louis, MO, USA) and 0.5 mg of DNase (Invitrogen, Mt Waverley, VIC, USA), bacterial cells were lysed and incubated on ice for 30 min and underwent centrifugation in an Avanti JXN-26 (Beckman Coulter, Mt Waverley, VIC, Australia) for 30 min at 15,000 RPM at 16° C to remove the cellular debris, and the resulting supernatant was filtered through a 0.45 µm low protein‐binding filter (Millipore, Merck, Darmstadt, Germany). Afterwards, this supernatant was injected into a 5 mL Ni^2+^ column (HisTrap HP, GE Healthcare, Chicago, IL, USA) on an AKTA pure FPLC in CAPS buffer A & B. The column was washed extensively with 10 column volumes of buffer to remove the contaminating endogenous proteins and eluted using a gradient elution buffer containing 500 mM imidazole. Finally, this protein was purified by size exclusion chromatography using a Superdex 200 column (GE Healthcare, Chicago, IL, USA) in glutathione *S*‐transferase (GST) buffer A containing 50 mM Tris and 125 mM NaCl (pH 8.0). Proteins that showed peak fractions were pooled and concentrated by an Amicon ultrafiltration device (Milipore, Merck, Darmstadt, Germany), and the purity of this Cap protein at different stages was assessed by sodium dodecyl sulphate–polyacrylamide gel electrophoresis (SDS-PAGE) (Fairbanks et al. 1971) stained with Coomassie blue for 5 min at room temperature (RT) with gentle shaking and de‐stained again in a solution containing 10% ethanol and 10% glacial acetic acid for at least 30 min at RT with gentle shaking. Finally, purified BFDV Cap protein was stored at −80° C at 1 mg/mL concentration in GST A buffer (pH = 8.0) until further downstream applications.

### Antigenicity test of Cap protein

Antigenicity of the purified Cap protein was confirmed by performing a western blot following the methods described by Patterson et al. (2013). Simultaneously, after SDS-PAGE, the gel was transferred to a nitrocellulose membrane (BioRad, South Granville, NSW, Australia) using freshly prepared transfer buffer (containing 11.64 g Tris Base, 5.86 g glycine, 750 μL 10% SDS, 400 mL methanol, and water to a final volume of 2 L) and a Criterion Blotter apparatus (BioRad, South Granville, NSW, Australia) at 80 V for 60 min. Afterwards, the membrane was blocked with Tris-buffered saline solution containing 0.05% Tween 20 (TBST) (Dako, Santa Clara, CA, USA) and 5% skim milk with gentle rocking for one hour at RT. The membrane was washed three times for 5 min each using TBST. It was then incubated for 2 h at RT, with similar gentle rocking as previously, with an anti-BFDV monoclonal antibody (MAb), 3F8-1 (1 mg/mL) (Ab Solutions, Perth, WA, Australia) (Shearer et al. 2008) diluted (1:800) in 5% skim milk-TBST solution. After that, the membrane was again washed thrice like before and incubated further for one hour, merged with horseradish peroxidase (HRP)-conjugated goat anti-mouse IgG (Sigma–Aldrich, St. Louis, MO, USA) diluted (1:1000) in 5% skim milk-TBST solution. The membrane was then washed three times and visualised by using 3,3′,5,5′-tetramethylbenzidine (TMB) Liquid Substrate System for membranes (Sigma–Aldrich, St. Louis, MO, USA) for 5 min. Finally, the colour development was stopped by washing the membrane with deionised water for 1 min.

### Selection of optimum antigen dilution

Before using the antigen, hemagglutination assays (HA) were performed using a twofold serial dilution of the purified BFDV Cap protein and BFDV VLPs using galah (*Eolophus roseicapillus*) erythrocytes according to the protocol developed by Raidal et al. (1993). Before proceeding to further downstream experiments, optimal concentrations of the recombinant Cap protein as well as the dilution of the DBS were determined by testing through the criss-cross (checkerboard) serial dilution (Fig. 2). Both positive and negative samples were assessed in different dilutions of dried blood spots and multiple concentrations of Cap protein. The dilution that showed the highest positive to negative ratio (PN ratio) has been selected for iELISA testing.

### HI test

Hemagglutination inhibition (HI) test of the samples has been done commercially in the veterinary diagnostic laboratory, Charles Sturt University. It has been operated simultaneously using recombinant BFDV Cap protein expressed in *E. coli* BL 21 (DE3) (Sarker et al. 2015b), according to the protocol developed by (Raidal and Cross 1994). HI titres are expressed as the reciprocal of the highest serum dilution that completely inhibits hemagglutination, indicating the presence of virus-specific antibodies. Titres of < 1:20 were considered undetected or negative, while positive results were recorded as serial two-fold dilutions (1:20, 1:40, 1:80, etc.), with higher titres indicating stronger antibody responses.

### Inter- and intra-assay precision

The assay’s reproducibility was assessed using HI positive and HI negative sera on different days under similar conditions. Intra-assay variability was evaluated by testing each sample in triplicate on a single plate. Each sample was analysed on three distinct plates to assess inter-assay variability. ELISA’s overall precision was defined by evaluating the variation between wells in the same test (intra-assay) or between different runs (inter-assay) by counting the mean optical density (OD), standard deviation (SD), and coefficient of variation (CV) as a ratio of the SD versus the mean OD value.

### iELISA procedure

This iELISA was developed and validated in compliance with the Office International des Epizootiques (O.I.E.) guidelines described in Jacobson (1998) to develop serological assays to diagnose infectious diseases. An in-house recombinant BFDV Cap protein (Sarker et al. 2015b) was used as the primary antigen to coat the 96-well Nunc-Immuno MicroWell plates (Sigma-Aldrich, St. Louis, MO, USA). The iELISA was operated following a slight modification of the previously described protocol (Das et al. 2026; Neef et al. 2024). One hundred microliters of the Cap protein, diluted with 0.05 M carbonate/bicarbonate binding buffer, was applied to coat each well, followed by an overnight incubation at 4 °C. The next day, the antigen solution was aspirated from each well, and wells were washed five times with tris-buffered saline containing 0.05% (v/v) Tween-20 (TBST). Wells were blocked with 100 µL TBST containing 5% skim milk powder for 1 h at 37 °C. This blocking was washed five times with TBST before adding 100 µL serum (primary antibody) and incubating at 37 °C for 1 h. After incubation, the primary antibody (Ab) was removed, and wells were again washed five times with TBST. 100 µL of Goat Anti-bird IgY H&L (Ab112773) conjugated with horseradish peroxidase (HRP) (Abcam, Waltham, MA, USA) diluted with TBST containing 5% skim milk (1:2000) (v/v) was then added and incubated at 37° C for 1 h, followed by removal of the secondary Ab solution and washing the wells five times with TBST. To develop the colourimetric reaction, 100 µL of HRP substrate solution (BioRad, South Granville, NSW, Australia) was added to the wells and incubated for 12 min at RT. This colour development is stopped by adding 100 µL of 0.18 M H_2_SO_4_. Optical density (OD) was recorded at 450 nm with a microplate reader (BMG Labtech, Mornington, VIC, Australia) using 630 nm as a reference. Four types of controls were used during each test: HI positive controls, HI negative controls, no primary antibody control, and no secondary antibody control.

### Data analysis

The diagnostic performance of iELISA was evaluated by comparing results with HI assay outcomes. Samples with HI tests having titres of < 1:20 were considered negative, and samples that showed HI titres > 1:20 (1:20, 1:40, 1:80, 1:160, 1:320, etc.) were considered positive. The mean OD value of each sample was counted for the analysis. To determine the cut-off points, both the HI and iELISA test results were analysed by a two-graph receiver operating characteristic (TG-ROC) (Greiner 1995; Greiner et al. 1995). The results were also plotted as a receiver operating characteristic (ROC) curve, and the area under the curve (AUC) was calculated to determine overall assay performance (Gardner and Greiner 2006). Gwet’s agreement was calculated to check the concordance between the iELISA and HI test (Gwet 2014). Data were analysed using RStudio version 4.3.2 (Team RC 2010).

## Results

### Antigenicity of BFDV Cap protein

**Fig. 1:**
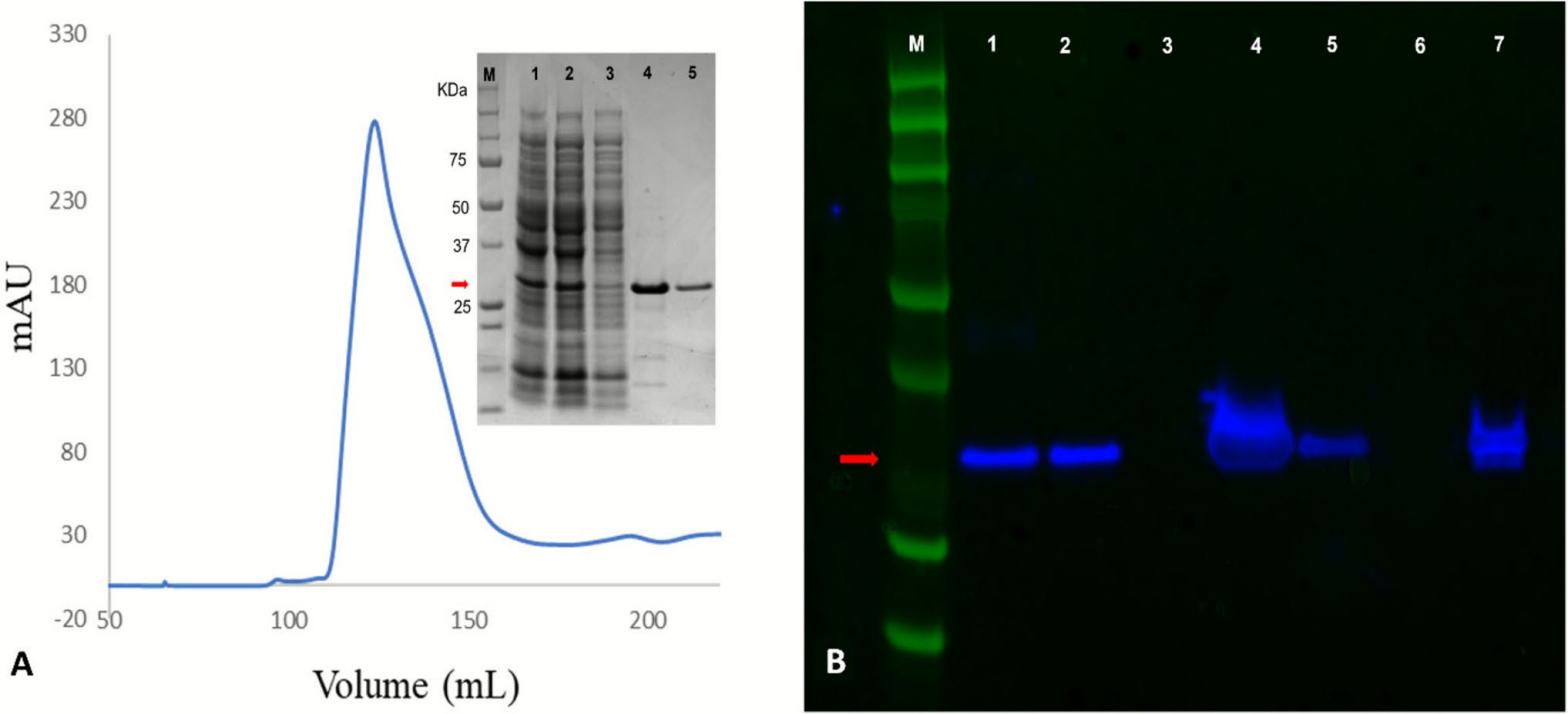
**A** Size exclusion chromatography profile and SDS‐PAGE of BFDV Cap protein showing the protein marker (lane M), whole *E. coli* cell lysate, soluble extract of *E. coli* protein fraction, flow‐through from the affinity column, affinity elution protein, and size exclusion chromatography elution of the protein in lanes 1 to 5, respectively; **B** western blot demonstrating antigenicity of BFDV Cap protein. M—protein marker, L1—whole *E. coli* cell lysate, L2—soluble extract, L3—flow‐through from the affinity column, L4—HIS affinity elution, L5—size exclusion elution of the BFDV Cap protein, L6—(negative control) lysozyme (1 mg/mL), L7—positive control

BFDV Cap protein was purified from other non-specific bacterial proteins using HIS and size exclusion chromatography, and subsequently visualised by SDS-PAGE analysis (Fig. 1A). In a concurrent test, the antigenicity of the yielded BFDV Cap protein was tested by western blot assay against a monoclonal BFDV antibody. Strong antigenicity of the BFDV Cap protein (Fig. 1B) in the test assures its potential to develop an iELISA using this antigen.

### Selection of optimum antigen dilution

**Fig. 2:**
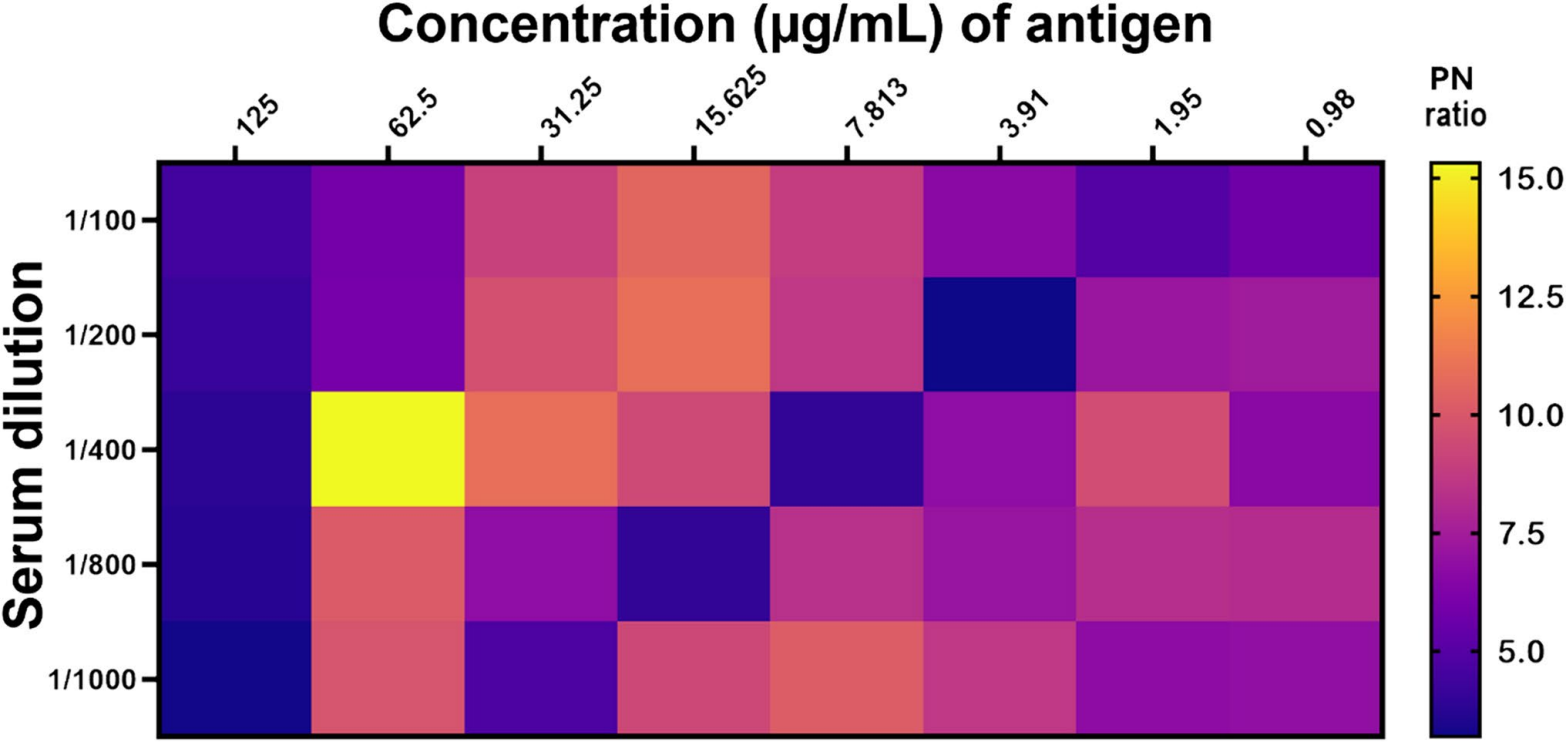
Checkerboard to identify the optimum dilution of the sample blood spot and optimum concentration of BFDV antigen. The optimal ratio of BFDV-positive to BFDV-negative OD value (PN ratio) is represented by different colours

Determination of the optimal concentration of the recombinant BFDV Cap protein to coat the microwell plate was a crucial step. We have optimised the concentration of this Cap protein along with the dilution of dried blood spots by using checkerboard dilution and concluded with the optimum Cap antigen concentration of 62.5 μg/mL, while the maximum dilution of the dried blood spots that showed the highest PN (Positive sample OD value/Negative sample OD value) ratio was 1:400 (Fig. 2); this means each blood spot was diluted in 400 μL of sterile PBS for serum extraction. In such concentrations of antigen and dilution of DBS, the PN ratio was 15.33, which was higher than any other combination. The optimised concentration of secondary antibody (Rabbit anti-bird IgY H&L) was 1:2000 (v/v) (Das et al. 2026; Neef et al. 2024).

### Inter- and intra-assay precision

The repeatability (intra-assay) of the developed protocol was verified using positive and negative samples with three replicates on the same plate. Reproducibility (inter-assay) was tested by using both positive and negative samples in three independent iELISA assays across three different batches. The inter- and intra-assay coefficients of variation (CV) for six control sera evaluated with the ELISA were all below 10% and within acceptable limits (Table 3). The mean inter-assay CV% was 3.97 (range 5.62–11.90), and the median intra-assay CV% was 2.81 (range 1.20–7.41) (Table 1). These were all within acceptable limits, indicating that the results were reproducible.

**Table 1:** Repeatability (intra-assay) and reproducibility (inter-assay) of iELISA

Sample	Intra-assay (OD)						Inter-assay (OD)					
	1	2	3	Mean	SD	CV %	1	2	3	Mean	SD	CV %
1	3.223	3.21	3.135	3.189	0.048	1.48	2.865	2.841	2.682	2.796	0.099	3.56
2	3.207	3.237	3.193	3.212	0.022	0.70	2.118	2.325	2.184	2.209	0.106	4.79
3	2.361	2.39	2.394	2.382	0.018	0.75	1.696	1.895	1.902	1.831	0.117	6.39
4	0.324	0.307	0.301	0.311	0.012	3.84	0.507	0.492	0.487	0.495	0.010	2.10
5	0.356	0.343	0.353	0.351	0.007	1.94	0.289	0.321	0.313	0.308	0.017	5.41
6	0.262	0.306	0.298	0.289	0.023	8.11	0.232	0.227	0.225	0.228	0.004	1.58
**Mean**						2.81						3.97

### iELISA and HI correlation

**Fig. 3:**
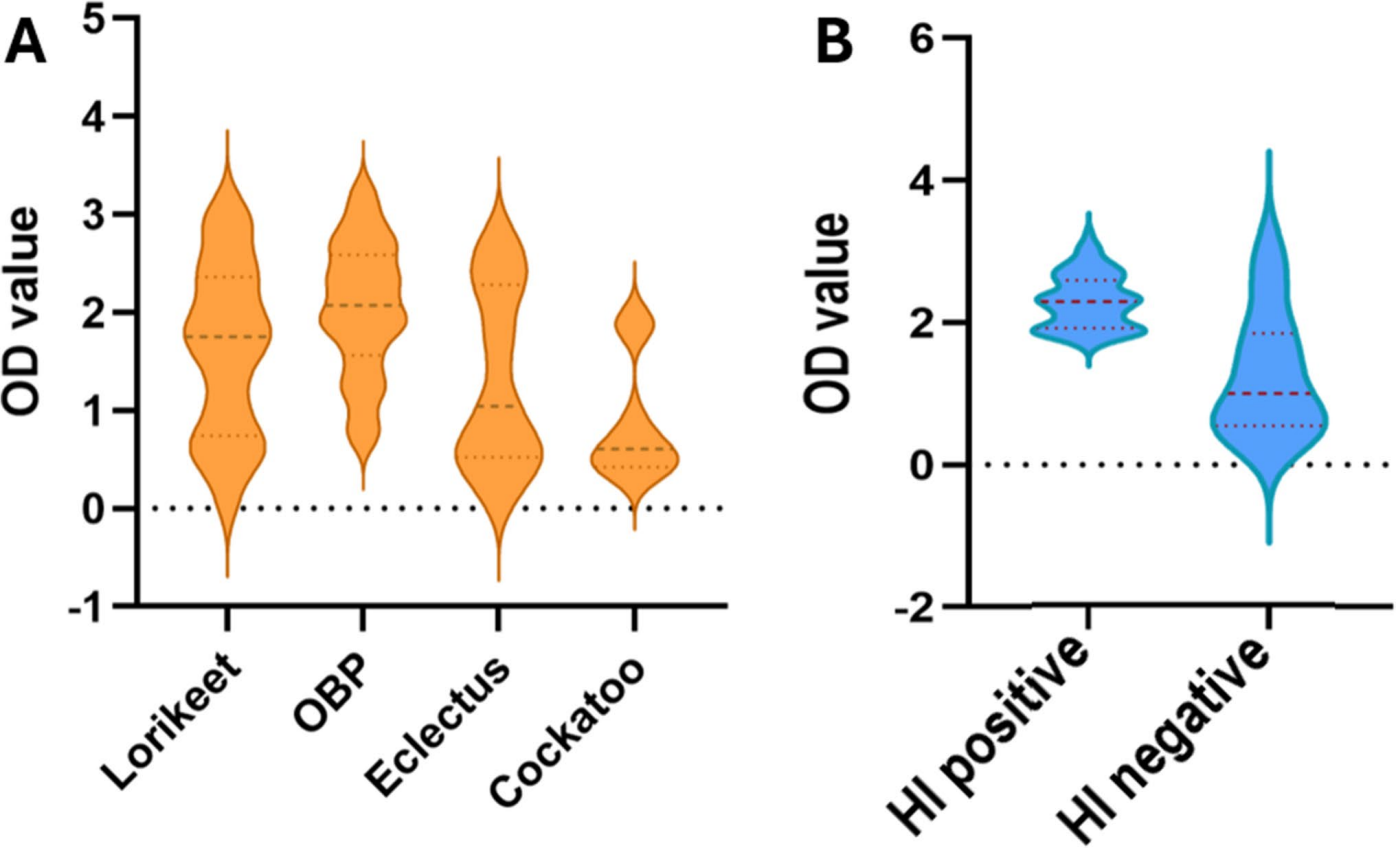
Violin plots showing the distribution of OD values in (**A**) Different species and (**B**) HI negative and HI positive DBS

Distribution of iELISA OD-values for four tested bird species has been shown in a violin plot (Fig. 3). Variability in OD values was observed between HI-positive and negative samples (Fig. 3B) as well as among the species (Fig. 3B), with Lorikeet and Orange-bellied parrot (OBP) exhibiting higher median values, suggesting a potentially stronger serological response to BFDV exposure. Outliers may be attributed to individual differences in immune response or variations in sample quality.

The differences highlight species-specific serological responses that warrant further investigation to optimise diagnostic accuracy across various avian hosts. The narrower interquartile range (IQR) in the positive sample plot in Fig. 3B indicates consistency in antibody detection among seropositive samples.

Conversely, HI negative samples showed lower iELISA OD values with a wider distribution of IQR, suggesting the presence of potential borderline cases or assay background reactivity, which indicates the presence of variable anti-BFDV antibody levels in those HI negative samples that cannot be detected by the HI test but can be differentiated well via the iELISA test. Diagnostic sensitivity and specificity of iELISA compared to HI test results have been charted in Table 2. The table shows that 85 samples out of 88 HI positive (96.59%) were also positive in the iELISA test, which reflects the highly sensitive property of the developed iELISA. A more interesting finding is that 36 samples out of 160 HI negative also became positive in the iELISA test, which may indicate early antibody development, detectable by iELISA but not by HI.

**Table 2:** Diagnostic sensitivity and specificity of the iELISA compared with the HI reference test

iELISA	HI		
	Positive	Negative	Total
Positive	85	36	121
Negative	3	124	127
Total	88	160	248
Coincidence	(85/88) = 96.59%	(124/160) = 77.5%	(209/248) = 84.27%

### iELISA cut-off point selection

**Fig. 4:**
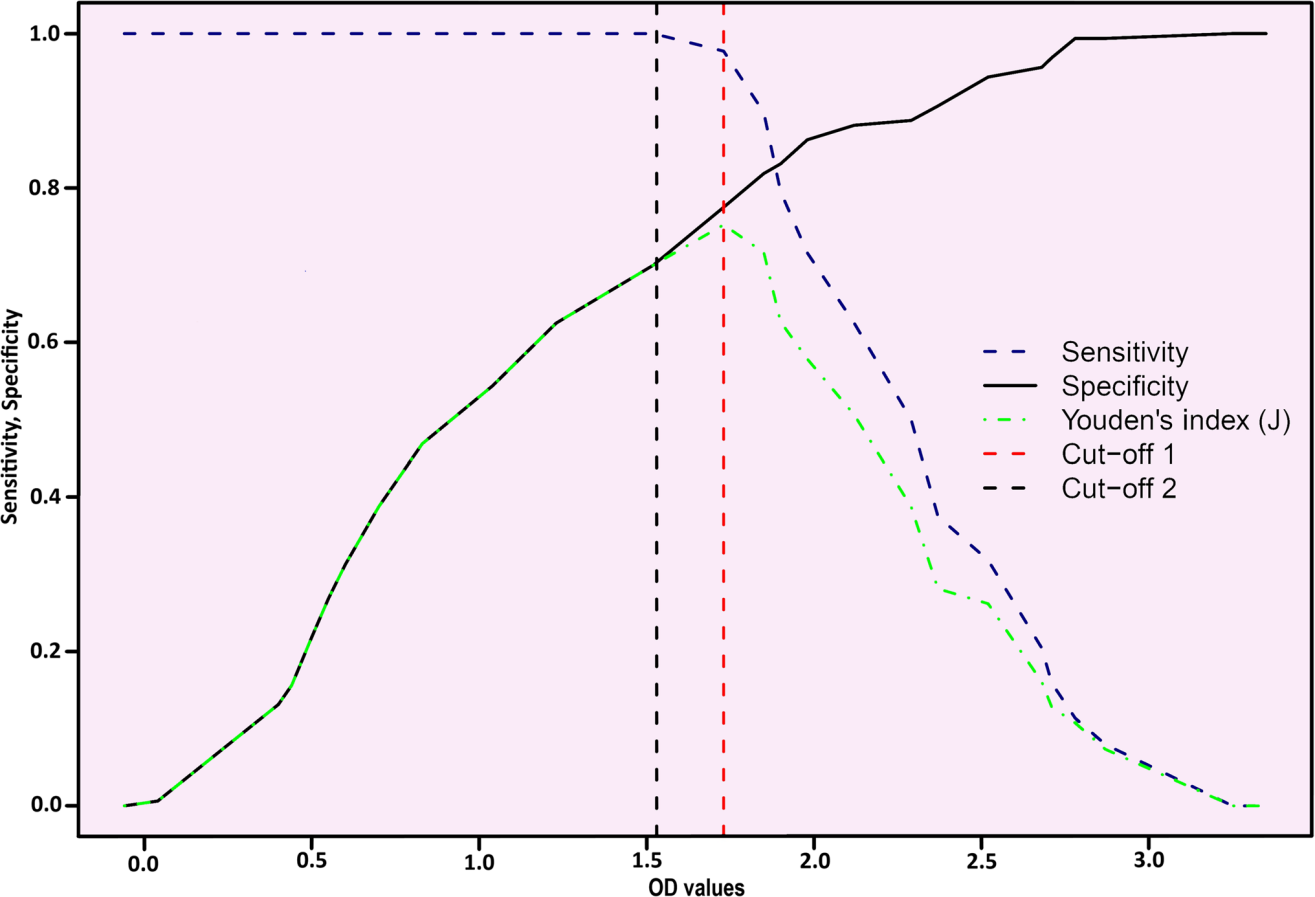
TG-ROC analysis of PBFD iELISA using the sensitivity (Se) and specificity (Sp) values. The optimal Youden’s index (J) was found to be at a cut-off of 1.73, with sensitivities and specificities of 96.5% and 77.5%, respectively. If the sensitivity is more important, the cut-off can be moved left to 1.55, where 100% sensitivity can be achieved

HI results were counted as the parameter to produce a TG-ROC curve. In HI testing, results are expressed as the reciprocal of the maximum serum dilution that completely inhibits hemagglutination, indicating the presence of virus-specific antibodies. In this study, the proportion of immune birds (HI titre ≥ 1:20) was 35.38%. TG-ROC curve is developed by plotting sensitivity (Se) and specificity (Sp) in the same plot to determine the cut-off value (Fig. 4). Equal Se and Sp (83%) can be found when the cut-off OD value is set to 1.88, but maximum Youden’s index depicts that the optimum cut-off point of this TG-ROC curve has to be 1.73. At this point, 96.5% sensitivity can be obtained. But if the sensitivity is the utmost priority, then the cut-off OD value can be shifted far left to 1.55 to ensure 100% sensitivity. Otherwise, different evaluation indices based on different cut-off values are shown in Table 3.

**Table 3:** Performance of iELISA based on different cut-off points. The optimal cut-off points and corresponding sensitivity, specificity, efficiency, and Youden's index values are highlighted in bold.

Cut-off points	Sensitivity (%)	Specificity (%)	Efficiency (%)	Youden’s index
1.55	100	71.6	81.6	0.72
**1.73**	**96.5**	**77.5**	**84.3**	**0.74**
1.88	83	83	83	0.65
2.34	42	90	72.5	0.318
2.57	25.8	95	70.5	0.21

### ROC evaluation

**Fig. 5:**
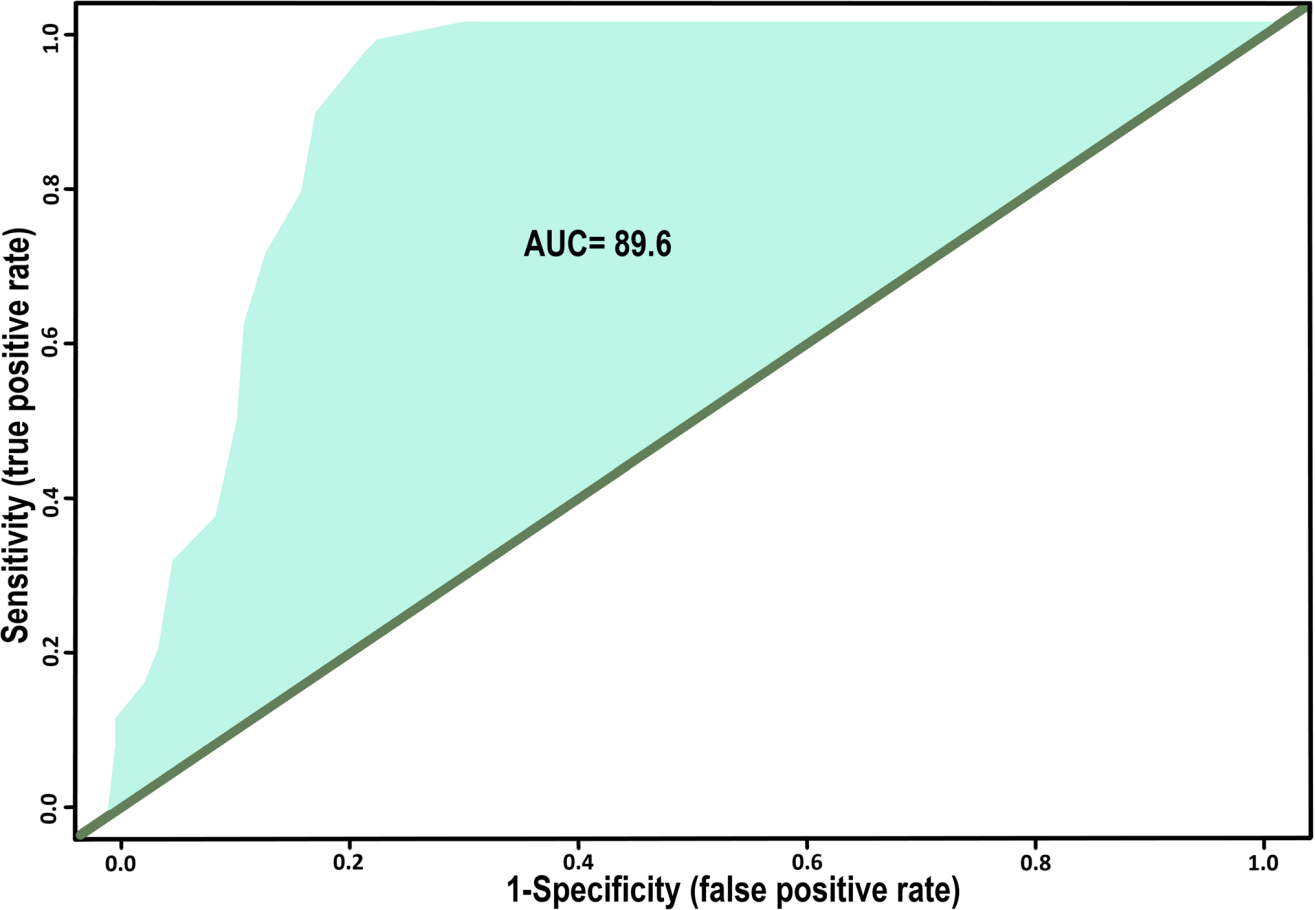
Receiver operating characteristic (ROC) curve analysis to illustrate the diagnostic performance of iELISA. Area under the curve (AUC) is 89.6% (95% CI 0.855 to 0.934)

The ROC curve evaluates the overall diagnostic ability of the test, plotting true positive rate (sensitivity) against false positive rate (1 − specificity). The curve in Fig. 5 demonstrates a high diagnostic accuracy, with an area under the curve (AUC) of 89.6%, with a 95% confidence interval (CI) ranging from 0.855 to 0.934. This indicates that the iELISA test has strong discriminative ability in differentiating between positive and negative cases and detecting PBFD antibodies.

### Inter-assay agreement

**Fig. 6:**
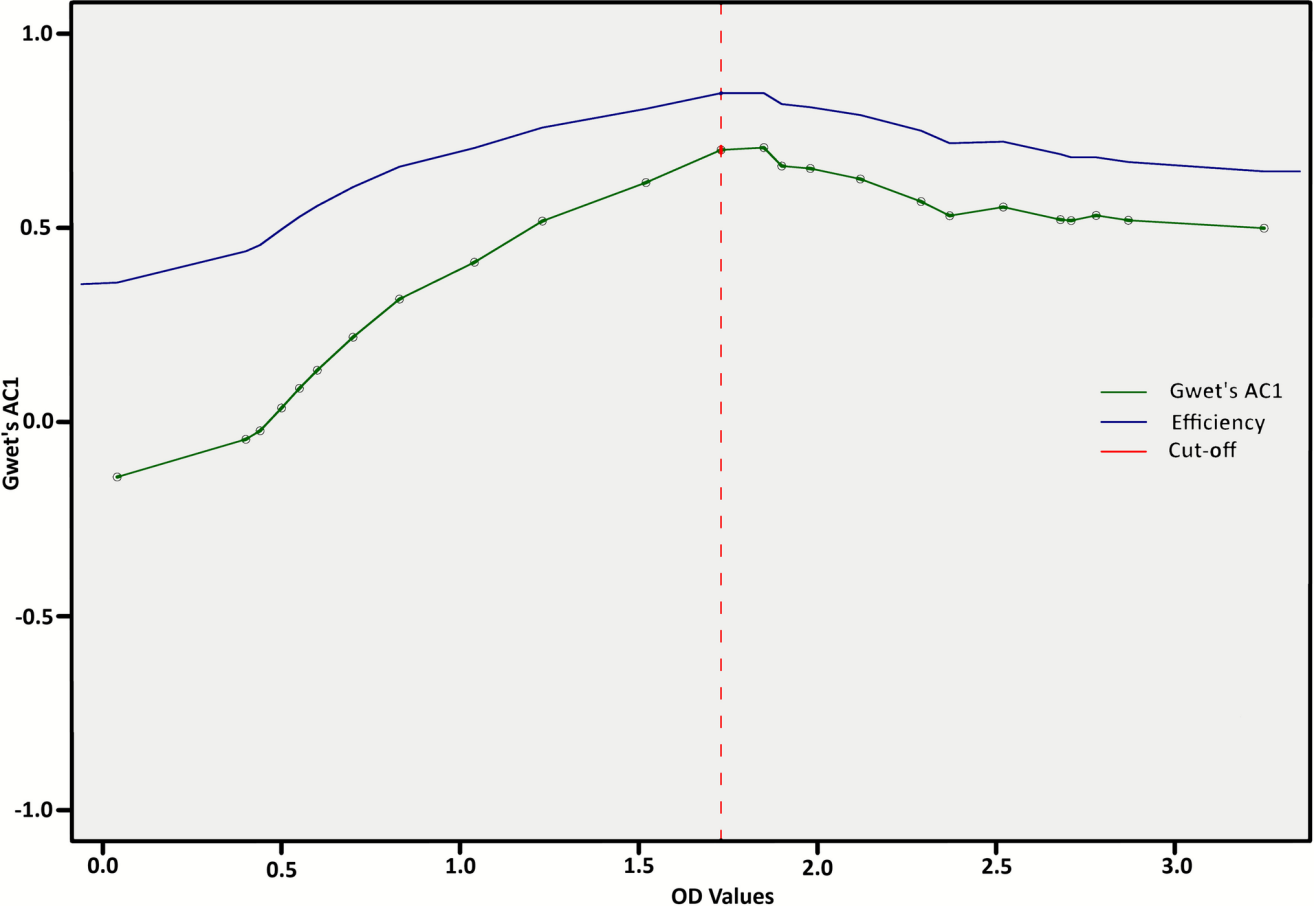
Gwet’s agreement coefficients against the OD values show a percent agreement of 0.843%, with the AC1 coefficient calculated at 0.69

To optimise the diagnostic threshold for the iELISA assay, Gwet’s AC1 (Agreement Coefficient 1) agreement coefficient and overall test efficiency (interpreted as diagnostic accuracy) were evaluated across a range of OD cut-off values. The concordance between the iELISA and the HI test was quantitatively assessed by calculating Gwet’s AC1 coefficient (Fig. 6), which serves as a robust measure of inter-rater agreement. The analysis yielded a percent agreement of 84.3%. The AC1 was calculated at 0.69 (Standard error 0.04), with a 95% confidence interval ranging from 0.602 to 0.783 and a *p*-value = 0, implying a highly statistically significant outcome. The AC1 coefficient’s magnitude demonstrates a substantial level of agreement, highlighting the consistency between the ELISA-derived classification and the reference HI results. The results suggest that the indirect ELISA, when analysed with the chosen optical density threshold, demonstrates significant diagnostic reliability and inter-method coherence.

## Discussion

ELISA is a well-established technique in both human and veterinary medicine for detecting viral infections and assessing immune status at the individual or flock level (Aydin 2015; Greiner and Gardner 2000; Matefo et al. 2022; Wright et al. 1993). Because BFDV infection in susceptible birds can manifest across a wide clinical spectrum, including asymptomatic shedders, there is a critical need for early and reliable diagnosis to limit transmission (Kim et al. 2024). We developed an iELISA using recombinant capsid VLPs expressed in *E. coli* to detect anti-BFDV antibodies, addressing the limitations of the existing HI assay, such as its dependence on specialised Galah erythrocytes (Raidal and Cross 1994; Raidal et al. 1993; Shearer et al. 2009a), which restricts broader laboratory usability. We also validated our iELISA against HI standards to establish it as a reliable, scalable, and standardised alternative for antibody detection in psittacine birds using DBS.

Previous ELISA methods for detecting BFDV antibodies have faced notable limitations; for example, Johne et al. (2004) used a truncated BFDV Cap and a secondary antibody specific for psittacine IgY, but their study included only 11 serum samples, limiting its representativeness. Besides, they used African grey parrots to produce the anti-psittacine IgY, which introduced uncertainty, as negative results in other species could reflect either a true absence of antibodies or insufficient cross-reactivity of the secondary antibody (Khalesi et al. 2005). These challenges underscore the need for a standardised, species-independent serological assay. We assessed our iELISA in at least 4 diverse groups of parrots and cockatoos, including eclectus parrots, lorikeets, sulphur-crested cockatoos, and orange-bellied parrots, thus providing a robust framework to assess the cross-species applicability of the developed iELISA.

The BFDV capsid (Cap) is the sole structural protein of the virion, serving multiple functions including forming the protective shell, binding viral DNA, and mediating nuclear transport essential for replication (Nath et al. 2021; Sarker et al. 2016). Importantly, the Cap protomers self-assemble into virus-like particles (VLPs), mimicking the biological structure of the wild-type virion as a *T* = 1 icosahedral particle. The retention of structural congruence across host-adapted BFDV genotype clusters, despite surface variations, suggests strong evolutionary constraints on capsid architecture (Das et al. 2019), which may explain the limited serotype diversity observed in extant parrots and underpins the feasibility of developing broadly applicable serological diagnostics. VLPs offer distinct advantages as antigens, as their repetitive and ordered surface array displays all relevant antigenic determinants, enhancing serodiagnostic sensitivity. In addition, their inherent thermostability and extended shelf-life compared to truncated proteins (Das 2018; Sarker et al. 2015b) further strengthen their suitability for reliable and scalable diagnostic applications. Collectively, these properties establish the BFDV Cap as an excellent antigen candidate for serological assays such as ELISA. Recombinant expression systems, including baculovirus and *E. coli*, have consistently demonstrated strong immunogenicity and antigenicity, with the *E. coli* system further optimised to produce stable, soluble, and antigenically valid protein (Johne et al. 2004; Khalesi 2007; Sarker et al. 2015b).

The optimisation of DBS dilution and BFDV Cap antigen concentration in this study was comparable to approaches reported in other contemporary works (Deng et al. 2018; Ge et al. 2018). Checkerboard titrations were employed to establish the optimal working conditions (Jacobson 1998), with results demonstrating that a DBS dilution of 1:400 combined with an antigen coating concentration of 62.5 µg/mL yielded the highest PN ratio (15.33%) (Fig. 2). The selected dilution aligns with values used in other avian serology studies (Deng et al. 2018; Ge et al. 2018; Liu et al. 2010; Neef et al. 2024; Wang et al. 2020; Xu et al. 2025) and reflects the need to balance adequate signal strength with minimal background reactivity. Lower dilutions often produced stronger optical density readings but were associated with increased background absorbance, whereas higher dilutions reduced nonspecific binding but risked signal loss in low-titre samples. The chosen 1:400 dilution, therefore, represents a robust compromise, maximising sensitivity while retaining assay specificity. Consistent with best practice, efficient elution of antibodies was enhanced by chopping DBS punches into smaller pieces prior to immersion in PBS, increasing surface exposure and facilitating the release of immunoglobulins.

The optimal coating antigen concentration was determined to be 62.5 µg/mL, a value comparable to recent ELISA studies employing recombinant circovirus VLPs (Mercatali et al. 2018; Neef et al. 2024). Use of recombinantly expressed antigen in *E. coli* increases the chances of bacteria-derived protein contamination in the downstream applications. This protein can lead to generate false positive results, especially when an anti-bird antibody is used as a secondary antibody source. To avoid such consequences, we used *E. coli* BL21 (DE3) during the transformation of BFDV cap and maintained a two-step purification of the recombinantly expressed protein, HIS chromatography followed by size exclusion chromatography. Robichon et al. (2011) showed that using *E. coli* BL21 (DE3) substantially minimises *E. coli-derived* protein contamination. A secondary antibody was used at a 1:2000 dilution, which provided strong signal intensity while minimising background (Das et al. 2026). To simplify production and broaden species coverage, a polyclonal Goat Anti-bird secondary antibody was selected. Previous work has shown that commercially available anti-chicken IgY antibodies exhibit substantial cross-reactivity with immunoglobulins from diverse avian taxa, including psittacine birds (Cray and Villar 2008). This broad reactivity underpins the suitability of anti-bird antibodies for wildlife serology, where species-specific reagents are often unavailable. Furthermore, anti-bird antibodies have been successfully applied in indirect ELISAs, where the selection of an appropriate cut-off is critical for interpreting ELISA results, as it establishes the threshold for classifying samples as positive, negative, or inconclusive (Matefo et al. 2022). Traditional approaches, such as setting the cut-off at two or three standard deviations above or below the mean, assume a normal distribution of test values in the target population, which may not always be valid. To overcome this limitation, TG-ROC analysis was employed to objectively identify the OD cut-off for the detection of several avian viral infections, including flaviviruses (Hofmeister et al. 2016), alphaviruses (Fassbinder‐Orth et al. 2016), and poxviruses (Ellison et al. 2014; Ha et al. 2013), further supporting their diagnostic reliability and versatility.

The violin plots presented in Fig. 3 illustrate distinct patterns of iELISA OD value distribution both across species and in relation to HI serostatus. Among species (Fig. 3A), lorikeets and OBPs exhibited consistently elevated OD values, whereas eclectus showed marginal and cockatoos demonstrated a comparatively attenuated and compressed distribution. These interspecific differences likely reflect variation in historical exposure, host susceptibility, and immunological responsiveness to BFDV, as previously documented in psittacine populations (Das et al. 2019; Sarker et al. 2015a). Figure 3B demonstrates a pronounced separation between HI positive and HI negative samples, with HI positive individuals displaying uniformly higher OD values and limited overlap with the negative cohort. The broader distribution of the HI-negative samples may indicate low-level or past exposure not detectable by HI, consistent with the higher analytical sensitivity typically attributed to ELISA-based antibody assays (Aydin et al. 2025; Shah and Maghsoudlou 2016). These findings underscore the capability of the iELISA to discriminate reliably between seropositive and seronegative individuals across diverse host species and support its suitability as a robust serological tool for BFDV surveillance and diagnostic applications.

Considering the HI titre as the parameter, the TG-ROC curve was developed to generate the cut-off value. TG-ROC analysis offers an adequate array of cut-off values to achieve Se and Sp for both parametric and nonparametric approaches. TG-ROC curve facilitates a quantitative serodiagnostic test when estimates of costs associated with false-positive and false-negative results are available (Greiner 1996). For both parametric and nonparametric methods, it provides high sensitivity and specificity, as demonstrated in a study on Newcastle disease virus detection in quail serum (Oliveira et al. 2007). While conventional methods like Mean ± 2SD are commonly used, they can be arbitrary. ROC curve analysis is widely accepted for optimising cut-off values and comparing diagnostic test accuracy (Sharma and Jain 2014). It allows for improved test performance by adjusting cut-off values based on clinical conditions. Alternative approaches, such as one based on the area under the ROC curve, have been proposed to define optimal cut-off values (Unal 2017). These methods aim to maximise sensitivity and specificity, crucial for accurate serological diagnosis of infectious diseases.

The diagnostic performance of the iELISA was evaluated using multiple complementary approaches to provide a robust assessment of sensitivity, specificity, and overall reliability. TG-ROC curve analysis enabled visualisation of potential cut-off values, capturing the trade-offs between sensitivity and specificity and revealing the intermediate range of OD values that are not discernible from conventional ROC or AUC plots. Youden’s index (J) was used to determine the optimum threshold for cut-off in the TG-ROC curve (Li et al. 2025). Defined as (Sensitivity + Specificity − 1), this index identifies the cut-off point that maximises the difference between the true positive rate and the false positive rate across all potential values. Selecting the point of maximum J ensures the selection of a single threshold, providing the best possible combined balance of test sensitivity and specificity for interpretation (Nahm 2022). At the optimum Youden’s index, the iELISA achieved high sensitivity (96.5%) with a cut-off of 1.73 and an assay efficiency of 74%, while specificity was comparatively lower (77.5%) (Fig. 4). This pattern likely reflects limitations of the HI reference test, including the presence of false negatives, as suggested by the wider distribution of OD values for HI-negative samples in the violin plots. Adjusting the cut-off for specific applications allows flexibility: prioritising maximal sensitivity (100%) for detecting all exposed birds requires lowering the cut-off to 1.55, whereas ensuring balanced sensitivity and specificity shifts the cut-off slightly higher to 1.88. The OD range of 1.55–1.73 can thus be considered an intermediate range, where antibody detection should be interpreted alongside clinical history and signalment, which is particularly critical for managing BFDV in vulnerable or endangered psittacine populations (Khalesi et al. 2005).

Complementary ROC analysis confirmed the overall discriminative power of the assay, with an AUC of 89.6% (95% CI 0.855–0.934) (Fig. 5), reflecting excellent ability to distinguish positive from negative cases (Greiner et al. 2000; Hajian-Tilaki 2013; Swets 1988). Concordance between iELISA and the HI test was further assessed using Gwet’s AC1 coefficient. Gwet’s AC1 is a chance-corrected measure of inter-rater agreement specifically used for nominal data classifications (e.g., Positive/Negative), which overcomes the prevalence-dependent paradoxes associated with Cohen’s kappa (Wongpakaran et al. 2013). The analysis demonstrated 84.3% agreement. The coefficient was estimated with a Standard Error (SE) of 0.04. The SE quantifies the precision of the AC1 estimate, reflecting the variability expected from sampling, and is used to construct the resulting 95% confidence interval (0.602 to 0.783). The *p*-value (= 0) confirms a highly statistically significant level of agreement, indicating strong agreement beyond chance and confirming the reliability of the iELISA classification (Fig. 6). Together, these analyses highlight the consistency and diagnostic reliability of iELISA as a highly sensitive and broadly applicable diagnostic tool, with clearly defined cut-offs and interpretive guidance for intermediate results. The combined use of TG-ROC, ROC/AUC, and AC1 metrics provides a comprehensive evaluation framework, ensuring accurate detection of anti-BFDV antibodies and supporting its application in surveillance and management programs across diverse psittacine species (Ohyama 2021; Xu and Lorber 2014).

Selection of an appropriate cut-off is critical for interpreting ELISA results, as it establishes the threshold for classifying samples as positive, negative, or inconclusive (Matefo et al. 2022). Traditional approaches, such as setting the cut-off at two or three standard deviations above or below the mean, assume a normal distribution of test values in the target population, which may not always be valid. To overcome this limitation, TG-ROC analysis was employed to objectively identify the OD cut-off that provides the most diagnostically balanced threshold (Greiner 1995). We identified an optimal cut-off value of OD 1.73 for the BFDV iELISA, which corresponded to a sensitivity of 96.5% and the highest diagnostic efficiency (84%) as determined by Youden’s index. Adjustments to the cut-off can be made depending on whether higher sensitivity or specificity is desired. OD values between 1.55 and 1.73 define an intermediate range (IR), within which results are considered suspect; values below and above this range are interpreted as negative and positive, respectively (Xu et al. 1997).

While the VLP-based iELISA showed strong concordance with the gold-standard HI assay, with high sensitivity and diagnostic efficiency, several inherent limitations of the iELISA format should be acknowledged. As indirect ELISAs rely on secondary antibody for signal detection, assay performance might vary due to species-dependent cross-reactivity, variation in total IgY concentrations, and background reactivity. These factors may alter OD values independently of true serostatus and may produce weaker signals in species with inherently low immunoglobulin levels (Walker and Crowther 2009).

BFDV infects a wide range of psittacine species, and spillover infections have also been detected in *Passeriformes, Coraciiformes, and Strigiformes* (Das et al. 2019; MacColl et al. 2024; Peters et al. 2014). Our validation included samples from only four psittacine species. Although the test performed robustly within this group, small but consistent differences in cut-off OD values were observed across species (Fig. 3A), and these differences may be more pronounced in untested hosts. This strongly suggests that a universal cut-off value is unlikely to be suitable for all species. Therefore, caution is warranted when interpreting results from new host taxa. Species-specific cut-off values must be established using a panel of known negative birds, as well as clinically positive and viraemic individuals of the same species, before applying the assay for diagnostic purpose. Such validation should be undertaken alongside the HI assay, which, despite its known limitations, has anecdotally provided reliable results across a wide range of host species in laboratories equipped with an HI test setup (Raidal and Cross 1994; Raidal et al. 1993).

The secondary antibody used in this iELISA is a commercially available anti-bird polyclonal antibody with known cross-reactivity across multiple avian taxa (Escandon et al. 2019; Fassbinder‐Orth et al. 2016). However, the relative intensity of cross-reactivity likely varies among species and can influence observed OD values. Comprehensive benchmarking of this anti-IgY reagent across different host species was beyond the scope of this study. Accordingly, establishing accurate cut-off values for any new species requires testing a sufficient number of HI-validated true negative and true positive samples, ideally as part of ongoing laboratory-level assay optimisation.

Finally, newly developed iELISA demonstrates clear advantages over existing serological methods for BFDV detection, including haemagglutination inhibition (HI) and blocking ELISA (bELISA). The current standard, the HI test, relies on the availability of unique haemagglutinating Galah erythrocytes (Raidal and Cross 1994; Raidal et al. 1993), is labour-intensive due to the need to maintain donor flocks, and presents additional challenges for large-scale application due to ethical considerations. In contrast, the iELISA is simple, cost-effective, and broadly applicable through the use of species-independent secondary antibodies, making it particularly suitable for wildlife and mixed-species surveillance. Compared to bELISA, which requires complex assay design, costly monoclonal antibodies, and inversely proportional signal interpretation, the iELISA utilises recombinant capsid VLPs that preserve the native antigenic structure and employs a pan-avian secondary antibody, allowing broader species applicability, high-throughput processing, and scalable deployment. Collectively, these features establish the validated iELISA as a scientifically robust and operationally feasible platform for broad-scale BFDV serosurveillance.

## Conclusion

The indirect ELISA developed using the recombinant capsid protein of BFDV demonstrates high accuracy, reliability, and reproducibility for detecting anti-BFDV antibodies across multiple psittacine species. This assay represents a viable and effective alternative to the existing haemagglutination inhibition test, providing a universal serological tool for routine diagnosis, surveillance, and monitoring of BFDV in endemic populations. Beyond diagnostics, the iELISA will facilitate vaccine development by enabling post-vaccination serosurveillance, distinguishing immunostatus between vaccinated and unvaccinated birds, and supporting the evaluation of immune responses. Additionally, it offers valuable insights into BFDV pathophysiology, including temporal changes in antibody levels, maternal antibody transfer, and their influence on infection and immunity. In the absence of a universal serodiagnostic tool for psittacine BFDV, this iELISA has the potential to become a cornerstone in BFDV serodiagnosis, driving a paradigm shift in research, clinical management, and broad-scale serosurveillance of beak and feather disease virus.

## Supplementary Information

Below is the link to the electronic supplementary material.

ESM 1 Supplementary Material 1 (DOCX 1.42 MB)

## Data Availability

Data are available from the corresponding author on reasonable request.
