# Correction: Virus-like particle (VLP)-based indirect ELISA (iELISA) for the detection of beak and feather disease virus (BFDV) antibodies

**DOI:** 10.1007/s00253-026-13765-6

**Published:** 2026-02-26

**Authors:** Pangkaj K. Dhar, Tridip Das, Babu K. Nath, Prabal Chowdhury, Andrew Peters, Jade K. Forwood, Shane R. Raidal, Shubhagata Das

**Affiliations:** 1 School of Agricultural, Environmental and Veterinary Sciences, Charles Sturt University, Wagga Wagga, NSW 2678 Australia; 2 Training Hub Promoting Regional Industry and Innovation in Virology and Epidemiology, Gulbali Institute, Charles Sturt University, Wagga Wagga, NSW 2678 Australia; 3 Biosecurity Research Program and Training Centre, Gulbali Institute, Charles Sturt University, Wagga Wagga, NSW 2678 Australia; 4 Asia Pacific Centre for Animal Health, Melbourne Veterinary School, Faculty of Science, The University of Melbourne, Parkville, VIC 3010 Australia; 5 Melbourne Veterinary School, Faculty of Science, The University of Melbourne, Werribee, VIC 3030 Australia

**Correction: Applied Microbiology and Biotechnology (2026) 110:33**

10.1007/s00253-025-13683-z

In the originally published version of this article, several author-submitted corrections were not implemented during the production process. These corrections pertain to the textual revisions, figure revisions and clarification of content intended to improve accuracy and consistency of the manuscript.

The article has now been corrected to reflect the author’s submitted revisions.

The original article has been corrected.

