# Supplementary material for: Virus-like particle (VLP)-based indirect ELISA (iELISA) for the detection of beak and feather disease virus (BFDV) antibodies
Source: Appl Microbiol Biotechnol. 2026 Jan 23;110(1):33. doi: 10.1007/s00253-025-13683-z (PMC12831674; doi:10.1007/s00253-025-13683-z)
Supplement: Supplementary file 1 — Supplementary Material 1 (DOCX 1.42 MB) [file 253_2025_13683_MOESM1_ESM.docx]

**Supplementary files**

**Title: Virus-Like Particle (VLP) Based Indirect ELISA (iELISA) for the Diagnosis of Psittacine Beak and Feather Disease (PBFD)**

Pangkaj K. Dhar^1,2*^, Tridip Das^1,2,3^, Babu K. Nath^3^, Prabal Chowdhury^4^, Andrew Peters^1^, Jade K. Forwood^1,2,3^, Shane R. Raidal^5^, Shubhagata Das^1,2,3^

^1^School of Agricultural, Environmental and Veterinary Sciences, Charles Sturt University, Wagga Wagga, NSW-2678, Australia

^2^Training Hub Promoting Regional Industry and Innovation in Virology and Epidemiology, Gulbali Institute, Charles Sturt University, Wagga Wagga, NSW-2678, Australia

^3^Biosecurity Research Program and Training Centre, Gulbali Institute, Charles Sturt University, Wagga Wagga, NSW-2678, Australia

^4^Asia Pacific Centre for Animal Health, Melbourne Veterinary School, Faculty of Science, The University of Melbourne, Parkville, VIC-3010, Australia

^5^Melbourne Veterinary School, Faculty of Science, The University of Melbourne, Werribee, VIC-3030, Australia

^*^Corresponding author: Pangkaj K. Dhar; pdhar@csu.edu.au

Orcid ID: 0000-0002-9595-9980

**Supplementary figure 1:** Uncropped SDS gel image in support of **Fig. 1 (A)**


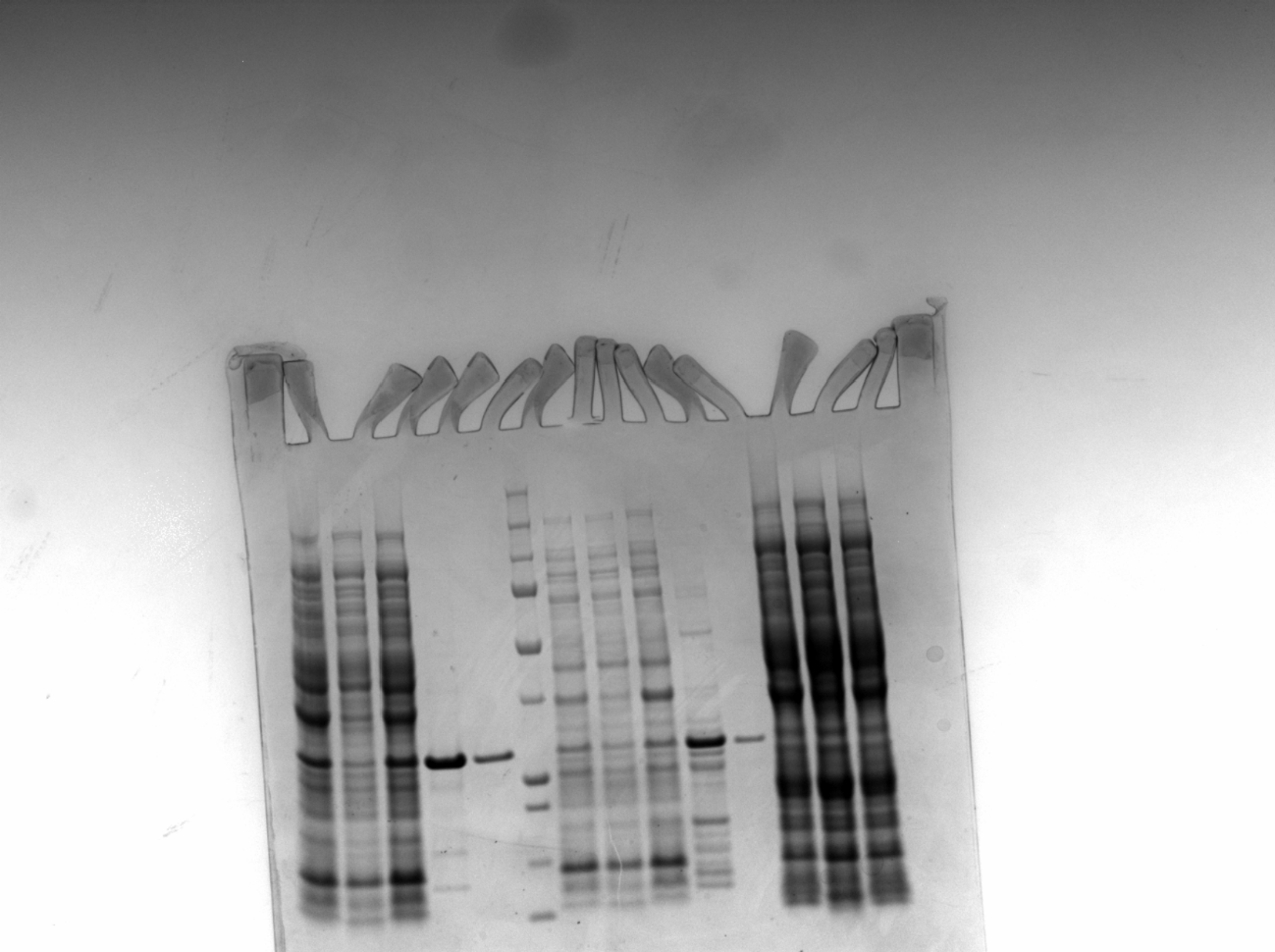


**Note:** Lane from the far left up to the marker on lane 6 have been cropped and used in the final manuscript. Lane 2 and 3 in this figure been swapped in the final manuscript figure to synch with the WB figure lanes of **Fig 1 (B)**, by representing same protein source in same respective lane. Marker have been switched to left in the final manuscript figure for better visualisation. Other lanes shown in the raw figure are not relevant to the manuscript.

**Supplementary figure 2:** Uncropped WB composite image in support of **Fig. 1 (B)**


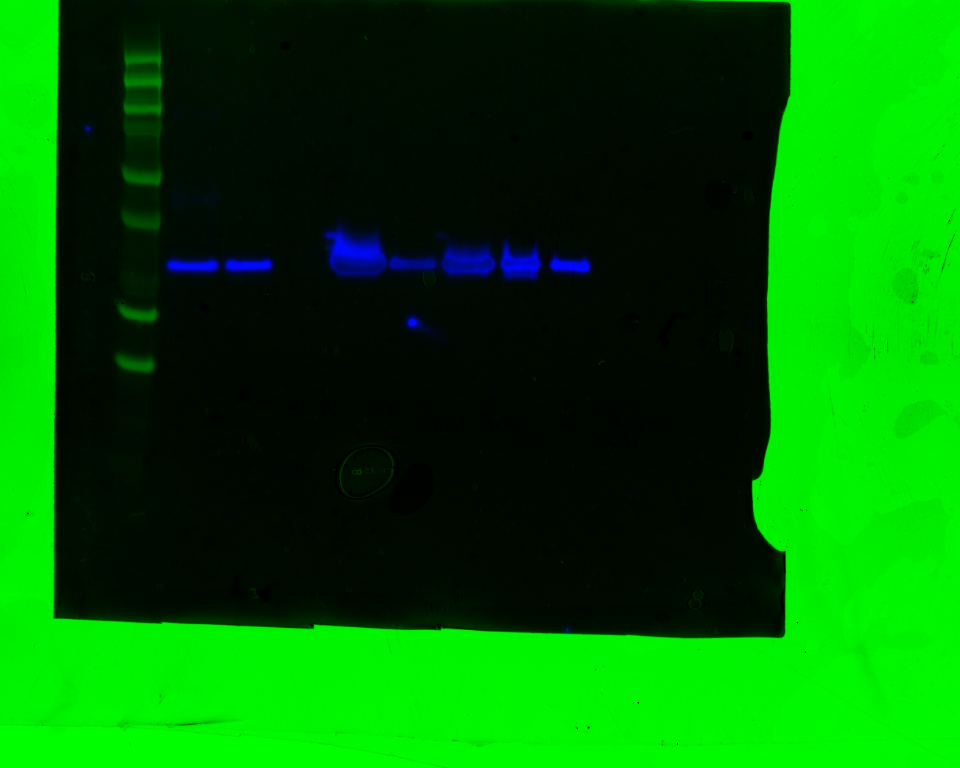


**Note:** This WB composite figure have been modified to be used in the manuscript. The whole WB is a part of vaccine and diagnostic tool development project. Both the capsid antigenicity and capsid powder (part of vaccine production) antigenicity have been tested and ran in the same gel to utilise resource. In this Composite figure, lane from left to right represent as following: L1- protein marker, L2- *E. coli* whole cell lysate, L3- soluble extract, L4- flow‐through from the nickel affinity column, L5-affinity elution, L6- size exclusion chromatography elution of the protein, L7- BFDV capsid without trehalose sugar (1mg/mL), L8- BFDV Capsid (1mg/mL) as positive control L9- spray-dried vaccine and phosphate buffered saline (1:10) and L10- lysozyme (1mg/mL) as negative control. Lanes irrelevant to the manuscript have been cropped and negative control (Lysozyme) lane have been swapped immediately before positive control for better visual representation.
